# Diet Quality, Measured by Fruit and Vegetable Intake, Predicts Weight Change in Young Women

**DOI:** 10.1155/2013/525161

**Published:** 2013-08-26

**Authors:** Haya M. Aljadani, Amanda Patterson, David Sibbritt, Melinda J. Hutchesson, Megan E. Jensen, Clare E. Collins

**Affiliations:** ^1^Priority Research Centre in Physical Activity and Nutrition, University of Newcastle, Newcastle, NSW 2308, Australia; ^2^School of Health Sciences, University of Newcastle, Newcastle, NSW 2308, Australia; ^3^Faculty of Nutrition and Health Science, King Abdul-Aziz University, Jeddah, MK 80200, Saudi Arabia; ^4^Faculty of Health, University of Technology Sydney, Sydney, NSW 2007, Australia

## Abstract

This study investigates the relationship between diet quality and weight gain in young women. Young women (*n* = 4,287, with 1,356 women identified as plausible subsample aged 27.6 ± 1.5 years at baseline) sampled from the Australian Longitudinal Study on Women's Health study completed food frequency questionnaires in 2003, which were used to evaluate diet quality using three indices: Australian Recommended Food Score (ARFS), Australian Diet Quality Index (Aus-DQI), and Fruit and Vegetable Index (FAVI). Weight was self-reported in 2003 and 2009. Multivariate linear regression was used to examine the association between tertiles of each diet quality index and weight change from 2003 to 2009. The ARFS and FAVI were significant predictors of 6-year weight change in this group of young women, while Aus-DQI did not predict weight change (*P* > 0.05). In the fully adjusted model, those who were in the top tertile of the ARFS significantly gained lower weight gain compared with the lower tertile for the plausible TEI sub-sample (*β* = −1.6 kg (95% CI: −2.67 to −0.56), *P* = 0.003). In the fully adjustment model, young women were classified in the highest FAVI tertile and gained significantly less weight than those in the lowest tertile for the plausible TEI (*β* = −1.6 kg (95% CI: −2.4 to −0.3) *P* = 0.01). In conclusion, overall diet quality measured by the ARFS and the frequency and variety of fruit and vegetable consumption may predict long-term weight gain in young women. Therefore, health promotion programs encouraging frequent consumption of a wide variety of fruits and vegetables are warranted.

## 1. Introduction

Recently, there has been a focus on evaluating the association between the nutritional quality of dietary intake and health outcomes [1]. Several studies have reported an inverse association between higher diet quality, all-cause, and chronic disease-specific mortality [1]. Our recent systematic review demonstrated a significant association between poor diet quality and greater weight gain [2].

A recent study demonstrated, in a nationally representative sample in the United States, that younger adults have poorer diet quality when compared with both children and older adults [3]. The evidence indicates that early adulthood is a high-risk period for weight gain, especially for females [4, 5]. For example, the Australian Longitudinal Study on Women's Health (ALSWH) data shows that when young women reach their forties, they will be heavier than middle-aged women are now [5]. However, our systematic review found limited studies that have specifically examined the association between diet quality and weight gain amongst young women [2]. Greater understanding of the association between diet quality and weight gain among young women may assist with the development of strategies for preventing weight gain during this life stage.

In this study we are analysing the relationship between three different approaches of diet quality indices including: index based on the food groups, which is the Australian Recommended Food Score (ARFS), and nutrients-based approach, the Diet Quality Index (DQI). In addition, we developed a new brief index that, based on consumption frequency and variety of fruits and vegetables items, is called the Fruit and Vegetables Index (FAVI). This tool can help to reduce the burden to both participants and researchers in terms of measuring diet quality. It can be used to predict weight change and therefore weight gain prevention or treatment interventions. Evidence suggests that greater consumption of fruit and vegetables in adults is associated with lower weight gain in longitudinal studies [6] and greater weight reduction in the intervention studies [6]. 

Notably, two studies exploring the association between diet quality and weight gain among middle-aged women have shown mixed results. A longitudinal study, conducted in an American middle-aged population, demonstrated that those who achieved the highest score on the DQI had a smaller weight gain (3 pounds) than those who achieved the lowest DQI score (5–8 pounds) during eight years of followup [7]. In contrast, we have previously demonstrated that overall diet quality measured using the ARFS did not predict weight gain in a sub-sample of middle-aged women from the ALSWH [8]. 

Therefore, the aim of this study was to investigate the relationship between diet quality and weight gain in young women from the ALSWH, using three different diet quality indices, ARFS, Australian-DQI (Aus-DQI), and the Fruit and Vegetable Index (FAVI). 

## 2. Materials and Methods

### 2.1. Subjects

**Figure 1:**
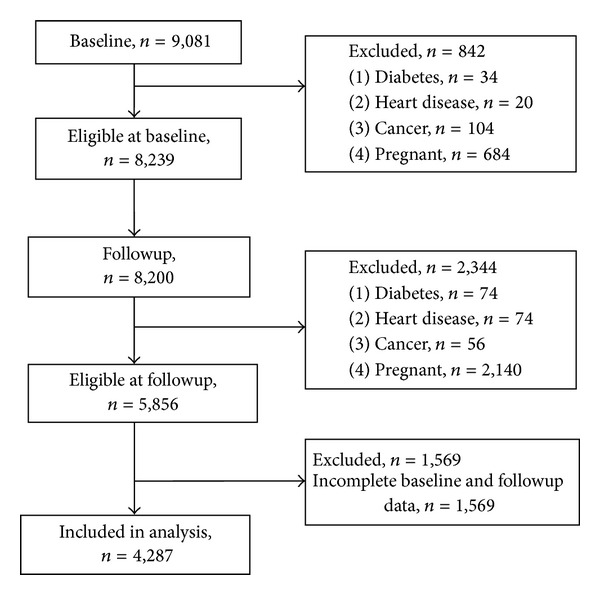
Flow chart of participant selection for analyses.

The population is a subset from the ALSWH cohort study. ALSWH recruited women into three cohorts according to age at baseline (young, middle-aged, and older). Further details of the cohort are published elsewhere [9]. Participants in the current analysis were drawn from the young women's cohort. Baseline (2003, aged 27.6 ± 1.5 years) and the six-year followup (f/u) (2009, aged 33.7 ± 1.5 years) were the two data time points selected for the current analyses. Participants were excluded if they had been diagnosed by a doctor as having diabetes, heart disease, or cancer (excluding skin cancer), or if they were currently pregnant. Of the 9081 young women at baseline, *n* = 8239 met the inclusion criteria. The response rate at followup totalled *n* = 8,200 young women, with *n* = 5856 eligible for inclusion. Complete baseline and followup data for weight, diet, and confounders were available for 4,287 women (Figure 1).

### 2.2. Anthropometry, Demographics, and Other Health Behaviours

Weight was self-reported at baseline (2003) and at followup (2009), in stones or kilograms (kg) to the nearest pound or gram, respectively. All data were converted to kilograms. Weight change (Δ) was calculated as the absolute difference (kg) in weight at followup from baseline. Participants self-reported their frequency of walking, moderate and strenuous physical activity (PA) [10]. There are two questions taken from the National Health Surveys which are validated and show reliability [10]. The questions were used to derive a PA score in metabolic equivalents (METs) per minute (METmins) at baseline. The total MET minutes were calculated as follows: (3 × minutes walking) + (4.0 × minutes moderate activities) + (7.5 × minutes vigorous activities) [11]. The cut points of PA were as follows: Nil/sedentary 0 < 40 MET minutes/week, low 40 < 600 MET minutes/week, moderate 600 < 1200 MET minutes/week, and high physical activity ≥1200 MET minutes/week. The highest qualification obtained was self-reported as “no formal qualifications,” “school certificate,” “higher school certificate,” “trade/apprenticeship,” “university degree or higher university degree.” Numbers of births were classified as: “no births,” “one to two births,” and “≥ three births.” The location of residence definitions used in the ALSWH dataset are taken from the ABS classifications. For this study, each region was classified as: urban (with 100,000 or more people), rural (with 200–999 people) and remote (<200 people). Relationship status was classified as “married,” “de facto,” “separated,” “divorced,” “widowed,” or “single.” Participants self-reported smoking status as “current smoker,” “never smoker,” or “ex-smoker.” The study was approved by the University of Newcastle and the University of Queensland Human Ethics committees and the current analysis on 13/10/2011 (EOI A342). 

### 2.3. Dietary Assessment

Baseline self-reported dietary intake was assessed using a food frequency questionnaire (Dietary Questionnaire for Epidemiological Studies Version 2 (DQES v2), Cancer Council of Victoria). The DQESv2 has been previously validated [12–14] and assesses intake of 74 food items over the past 12 months. Usual consumption frequency of each food item is indicated on a ten-point Likert scale, ranging from *“never*” up to *“three or more per day*.*”* Additional questions assessed the total number of daily serves of fruit, vegetables, bread, dairy products, eggs, fat spreads, and sugar, as well as the type of bread, dairy products, and fat spreads used. Nutrient intakes were computed using a food composition database of Australian foods (NUTTAB 1995, Australian Government Publishing Service, Canberra, Australia) and software developed by the Cancer Council of Victoria. 

#### 2.3.1. Australian Recommended Food Score (ARFS)

The ARFS is a food-based index adapted to the Australian adult population by Collins et al. (2008) [1] from the original US version of the Recommended Food Score by Kant et al. [15]. The optimal ARFS reflects greater adherence to Dietary Guidelines for Australian Adults [16]. The ARFS ranges from zero to a maximum score of 74, with a higher score indicating greater diet quality. The seven subscales with different maximum points include vegetables (22 points), fruits (14 points), protein foods (14 points), grains (14 points), dairy (seven points), fats (one point), and alcohol beverages (two points) [1]. Each food item is scored as one or zero, with an additional score for food quality. Scoring is independent of reported amounts of food, such that items consumed less than once a week scored zero and those consumed once a week or more scored one. More details about the scoring methods and items of the ARFS can be found in the Supplementary Material (see Appendix 1 in Supplementary Material available online at http://dx.doi.org/10.1155/2013/525161).

#### 2.3.2. Australian Diet Quality Index (Aus-DQI)

The DQI was chosen as studies have shown that higher scores on this index are associated with lower weight gain [7]. A longitudinal study, conducted in a middle-aged US population, demonstrated that those who achieved the highest Diet Quality Index (DQI) scores had a smaller weight gain (3 pounds) than those who achieved the lowest DQI score (5–8 pounds) after eight years of followup As part of the adaptation of the US DQI to the Aus-DQI, the scoring was adjusted to incorporate the Australian Nutrient Reference Values (Aus NRVs) [16, 17]. The original DQI was designed to evaluate adherence to the fourth edition of the Dietary Guidelines for Americans [18], and each participant achieved one point for each of the following nutrients: “total fat (<30% kcal), saturated fat (<10% kcal), cholesterol (<300 mg/d), sodium (<2400 mg/d), and carbohydrate (>50% kcal)” [7]. The Aus-DQI was adapted to Australian recommendations. However, given that there is currently no Australian recommendation for the intake of cholesterol, this subscale was omitted. In the Aus-DQI, each participant gets a maximum of one point for each of the four sub-scales: total fat <35% kJ, saturated fat ≤7% kJ, carbohydrate ≥45% kJ, and sodium <2300 mg/d. These targets were set according to Australian and New Zealand Nutrient Reference Values [17]. The total Aus-DQI score ranges from zero to four points. 

#### 2.3.3. Fruit and Vegetable Index (FAVI)

Evidence suggests that greater consumption of fruit and vegetables in adults is associated with lower weight gain in longitudinal studies [6] and greater weight reduction among overweight and obese participants in the intervention studies [6]. Fruit and vegetable consumption data, derived from the baseline DQESv2, were used to inform the development of the FAVI. The FAVI is divided into two sub-scales: the fruit sub-scale, which contains 13 items, including canned or frozen fruit and fruit juices, and 11 types of fresh fruit, such as oranges, apples, and pears, and the vegetable sub-scale which contains 24 items, including potatoes cooked without fat, tomato, zucchini, mushroom, celery, and beans. Consumption frequency of all fruit and vegetable items was scored using the full range of the FFQ Likert scale from zero to nine, with “never” scored as zero and “≥3 times per day” scored as nine points. In the FAVI score zero point are awarded for those who consume no items of fruit and vegetables. One point is awarded for consuming each fruit or vegetable item less than once per month, two points for one to three times per month, and three points for once per week, with an additional point awarded on an increasing scale for each additional frequency response category up to a maximum of nine points for consuming an item three or more times per day. The maximum possible score is 117 for the fruit sub-scale and 216 for the vegetable sub-scale, giving a maximum total FAVI score of 333 points. A higher FAVI score indicates a greater variety and frequency of usual fruit and vegetable consumption. 

### 2.4. Statistical Analysis

Data were assessed for normality and presented as means and standard deviations. Results were considered statistically significant if *P* < 0.05. Weight and macronutrient variables were treated as continuous variables. Each dietary quality index was categorised into tertiles based on the distribution of the total number of participants included in the study, to give approximately equal numbers in each tertile. For each diet quality index, data between the tertiles were compared using ANOVA. Multivariate linear regression was used to predict six-year weight change (95% confident interval, *P* value). The diet quality index of interest was the independent variable(s), with the first tertile being the reference value. To address misreporting and try to identify the subgroup least likely to have under- or over-reported total energy intake; the ratio of energy intake (EI) to basal metabolic rate was calculated. Basal metabolic rate (BMR) for each woman was calculated using the Schofield equations [1]. Using the Goldberg equations for a moderate physical activity level of 1.55 for this group then a TEI of 1.27–2.1 times BMR can be considered plausible [19, 20]. Three different regression models were applied to both the total sample and the subsample with plausible total energy intakes: (1) crude model: unadjusted; dependent variable = Δweight; independent variable = baseline diet quality index of interest. (2) The second model is adjusted specifically for the most important covariates that were available in the ALSWH data set, the specifically adjusted model: adjusted for physical activity, education, number of births, location of residence, marital status, smoking, and weight at baseline. (3) The final model: sought to evaluate the impact of energy intake on the model and included all the co-variates as per model 2 above, but also included total energy intake (TEI). All statistical analyses were carried out using STATA (version 11.1 for windows, 2009, StataCorp LP, USA).

## 3. Results

### 3.1. Subject Characteristics

The total number of women included in this analysis, with complete baseline and followup data on weight change and diet, was *n* = 4,287.  Table 1 summarises subject characteristics at baseline and weight change. Overall, the mean weight change from 2003 to 2009 was +3.6 ± 1.5 kg. A comparison of diet quality scores and co-variates for those with and without complete data on weight change from 2003 to 2009 indicated that there were no differences in diet quality score, measured by all three indexes: education, PA, and smoking status, *P* > 0.05 (data not shown). For those who had missing data on FFQ, they also had missing data on the other co-variates.

### 3.2. Weight and Macronutrients Across Diet Quality Index Tertiles

There was no significant difference across tertiles of ARFS for mean weight change, but there were significant differences in the means of energy intake (kJ/d), fibre (g/d), carbohydrate (%), and protein (%) intakes total fat (%) and saturated fat (%) intakes observed across ARFS tertiles (Table 2). In the plausible TEI sub-sample, the top tertile of ARFS had the lower mean weight gain (2.9 ± 7.9) kg; however, this was not significantly different compared to the second and lowest tertiles (3.4 ± 7.7 kg and 4.0 ± 7.9 kg, resp.). The top tertile of ARFS had greater total energy intake (TEI) (kJ/d), fibre (g/d), carbohydrate (%) and protein (%) intakes and lower total fat (%) and saturated fat (%) intakes compared with other tertiles (Table 2). 

There was no significant difference in the mean weight change across the Aus-DQI tertiles (Table 3). Aus-DQI tertile 3 had lower TEI (kJ/d), fat (%), saturated fat (%), protein (%), and fibre (g/d) intakes and higher carbohydrate intakes (%), compared with the other Aus-DQI tertiles (Table 3). In the plausible TEI sub-sample, there was no significant difference in weight changes between tertiles of Aus-DQI. There were significant differences in means of carbohydrate (%), fiber (g/d), energy intake (kJ/d), total fat (%), saturated fat (%), and protein (%) intakes.

There was a significant difference in mean weight change across the FAVI tertiles (*P* = 0.003), with the third tertile of FAVI gaining the least amount of weight during the six years of followup compared with the other tertiles (Table 4). The intakes of fat (%) and saturated fat (%) were significantly lower, while TEI, protein (%), carbohydrate (%), and fibre (g/d) intakes were significantly higher in the third FAVI tertile. In the plausible TEI sub-sample, those in the lower tertile of FAVI had significantly greater weight gain than those in the second and the top tertiles of ARFS (Table 4). In the plausible TEI, the top tertile of FAVI had lower total fat (%) and saturated fat (%) but greater intakes of carbohydrate (%), Fiber (g/d). There were no significant differences between TEI and protein intake across FAVI tertiles. 

### 3.3. Baseline Diet Quality Indices as a Predictor of Six-Year Weight Gain

In the plausible TEI sub-sample, only those in the top tertile of the ARFS had significantly less weight gain (1.6 kg) compared with those in the lower tertile of the ARFS. In the fully adjusted model, those who were in the top tertile of the ARFS had significantly lower weight gain compared with the lower tertile for the plausible TEI sub-sample, (*β* = −1.6, CI: −2.67 to −0.56, *P* = 0.003). 

Baseline FAVI was a statistically significant negative predictor of weight gain in this group of young women, while ARFS and Aus-DQI were not statistically significant predictors of weight change (Table 5). Compared with the first tertile of FAVI, women in the third tertile had the lowest weight gain over six years (*β* = −0.72, CI: −1.4 to −0.03, *P* = 0.04) in the fully adjusted model. 

In the plausible TEI sub-sample, we found that those in the second and third tertiles of FAVI had significantly less weight gain compared with the first tertile. More specifically, we found that, in the fully adjustmed model, those who were in the top tertile of FAVI gained the lowest weight compared with other tertiles (*β* = −1.6, CI: −2.4 to −0.3, *P* = 0.01). The second tertile of FAVI: (*β* = −1.5, CI: −2.4 to −0.2, *P* = 0.02), also had lower weight gain than the first tertile. 

## 4. Discussion

The current study tested three different diet quality indices as predictors of weight change over the subsequent six-year period in a cohort of young women participating in the ALSWH. it demonstrated that higher scores on either a food variety and frequency index (ARFS) or an index based on fruit and vegetable variety and frequency alone (FAVI), predicted lower six-year weight gain in this group of women. In the whole sample the ARFS showed no relationship with prospective weight gain, while the Aus-DQI showed no relationship in either the whole or the plausible TEI sub-sample. 

The main findings of this study support the role of increased fruit and vegetable consumption as a key strategy to prevent weight gain, particularly for young women. This is consistent with a recent prospective study by Vioque et al. (2008) [21] among 206 healthy Spanish adults aged 15 to 80 years. Vioque et al. found that those in the highest quartile of vegetable and fruit consumption (>698 g/d) at baseline, as assessed by a FFQ, had a reduced risk of weight gain (≥3.41 kg) compared with those who were in the lowest quartile of vegetable and fruit consumption during 10 years of followup (OR 0.22, 95% CI 0.06 to 0.81, *P* trend = 0.022). Another prospective study conducted by Kahn et al. (1997) [22] in 79,236 healthy white non-Hispanic American adults found that greater consumption of vegetables (highest quintile) was associated with a smaller gain in BMI over 10 years of followup (*β* = −0.12; *P* = 0.09, 0.012) for women and men, respectively. A systematic review of experimental studies supports increasing fruits and vegetables to support weight management [23]. A randomised controlled trial in 97 obese adults aimed to assess the effect of two approaches to weight loss, a decreased dietary fat intake, or an increased intake of fruit and vegetables plus decreased dietary fat intake over one year (both groups reduced fat by the same amount) [24]. The main finding demonstrated that those who increased their consumption of fruit and vegetables and decreased dietary fat achieved significantly greater weight loss, 7.9 ± 0.9 kg compared with 6.4 ± 0.9 kg for the other group [24]. A trial carried out in Brazil [25] in 80 overweight people found that those who increased their fruit and vegetable intakes by 100 g/d experienced lower weight gain (300 g cf. 550 g) over six months compared with those who did not change their intakes for fruits and vegetables. In the whole sample, we found that higher TEI was associated with the highest FAVI score. However, we also found that higher FAVI scores were associated with the lowest weight gain. However, in the plausible TEI sub-sample, there was no significant difference between TEI across the tertiles of FAVI, as shown in Table 4. One possible explanation for this is that there are only a limited number of energy-dense, nutrient-poor foods in the FFQ, meaning that TEI from these items may not be well captured. Those with a lower TEI may have higher energy intakes from these non-FFQ items. Although the ARFS and FAVI were strongly positively correlated with each other; the ARFS in the full sample did not predict weight gain, while FAVI did in both the whole and plausible energy intake samples. This suggests that neither the ARFS nor the FAVI captures the association between foods that are energy dense, nutrient poor, and weight change. In the current study, the focus was to examine the association between the healthful, nutrient-dense food items, and weight change. Higher diet quality index scores have been shown in a review to predict the risk of future morbidity and mortality [1].

The Aus-DQI failed to predict weight gain during the followup period in this sample of young women, even though it incorporates sub-scales for the percentage energy from total fat, saturated fat and carbohydrate intakes, and total sodium intake. The limited scoring scale and that it had not been previously validated limit the interpretation of this result. This also may be due to the limited list of energy-dense, nutrient-poor foods, particularly soda, and other sweetened beverages within the DQES which is to be expected given that it was developed more than 20 years ago. Thus, an assumption and limitation are that TEI may be partly underestimated due to the items in the FFQ. In the whole population,we found that the lowest intake of fiber across the Aus-DQI tertiles was for the top tertile, or highest diet quality scores. Among those women with plausible TEI however, we found that the highest Aus-DQI tertile was associated with higher intakes of fiber. This difference is likely due to misreporting of TEI and we expect that the results in the plausible TEI sub-sample are more likely to be more accurate. 

The ALSWH cohort is a representative sample of the population of Australian women, and the weight change data from the current study indicate that weight gain is common among young women. In addition, very few young women achieved a high diet quality score. The mean diet quality score in the highest tertile of each index was not high, indicating that interventions seeking to optimise diet quality in this age group are warranted as has been suggested previously [26–28]. In addition, a recent systematic review [29] has highlighted that intervention studies specifically targeting body weight are needed to prevent the development of overweight and obesity in this age group. 

There are a number of major limitations that need to be addressed. This includes that there are a large number of women with missing data on weight or dietary intake at baseline and follow-up. Furthermore, a limitation that needs to be acknowledged is loss to follow up. In the ALSWH study, attrition is the most common in participants with a lower education, those not born in Australia and those with poorer health or who smoke [30]. The potential impact of this attrition is that there may be selective loss of those whose weight change is greater and/or have poorer dietary intake than in those who have been retained. This potentially underestimates the ability of diet quality indexes to detect a relationship between dietary patterns and weight change. In addition, dietary intake was only measured once over this time period, and we are therefore not able to evaluate how or whether the women changed their eating habits over time. 

Furthermore, all data were self-reported including weight which introduces a potential reporting bias. A previous validation study of self-reported weight on mid-aged women from the ALSWH demonstrated that there was no clinical difference between self-reported weight and measured body weight [31]. While a similar validation has not been done for the young cohort of the ALSWH, it might be expected to give similar results. Another limitation that must be considered is that the Aus-DQI was not validated but was adapted from the original USA DQI which was based on American NRVs not Australian NRV's. As a consequence, results should be interpreted with caution.

The strengths of this study include the use of a healthy representative sub-sample derived from ALSWH population, with an adequate followup period. In addition, we used appropriate and rigorous statistical analyses and three different approaches to the measurement of diet quality to reflect the National Dietary Guidelines for Australia, including two based on established methods and one new index based only on fruit and vegetable intakes. This new tool provides a simple approach to diet quality assessment and successfully predicted weight change in this cohort of young women. Further research evaluating and validating the performance of FAVI in other age and gender groups is warranted. 

## 5. Conclusion

Frequency and variety of fruit and vegetable intakes, and overall diet quality predicted weight gain over six years in this healthy population group of young women. Strategies to encourage young women to more frequently consume a greater variety of fruit, and vegetables are required and may assist to prevent weight gain in this age group.

## Supplementary Material

Details of items, scoring methods and ARFS subscales can be viewed online in Appendxi: 1 of the Supplementary Material associated with this manuscript.

## Figures and Tables

**Table 1:** Demographic characteristics of young women in the Australian longitudinal study on women's health (ALSWH) (*n* = 4,287) at baseline (2003) and followup (2009).

Characteristic	Baseline		Follow-up	
	Total sample (*n* = 4,287)	Valid TEI (*n* = 1,356)	Total sample (*n* = 4,287)	Valid TEI (*n* = 1,356)
*Anthropometry *				
Obesity (%)	15.7	11.6	20.6	16.7
Overweight (%);	22.5	19.6	25.0	23.6
BMI; mean ± SD	24.8 ± 5.5	23.9 ± 4.9	26.2 ± 6.0	25.2 ± 5.5
Weight (kg); mean ± SD	68.3 ± 15.8	66.0 ± 14.1	71.7 ± 17.4	69.4 ± 15.5
*Diet quality index scores *				
ARFS	29.5 ± 9.2	31.4 ± 8.8	n/a	n/a
Aus-DQI	1.4 ± 0.8	0.7 ± 0.9	n/a	n/a
FAVI	78.0 ± 39.7	94.1 ± 26.9	n/a	n/a
*Demographics *				
Age (years); mean ± SD	27.6 ± 1.5	27.7 ± 1.5	33.7 ± 1.5	33.8 ± 1.5
Total energy intake (kJ); mean ± SD	6980.7 ± 2921.1	8975.3 ± 1386.3	n/a	n/a
Physical activity in METs (nil/low/moderate/high); (%)	8.9/35.3/22.8/33.0	8.9/9.7/20.4/31.1	n/a	n/a
Smoking status (never/ex-smoker/current); proportion (%)	58.7/18.3/23.0	60.1/16.5/33.4	n/a	n/a
Residence (urban/rural/remote); proportion (%)	57.3/39.0/3.7	55.3/41.0/3.7	n/a	n/a
Highest education (nil/school certificate/trade/university degree); proportion (%)	1.5/31.0/3.3/64.3	1.0/29.9/3.1/66.0	n/a	n/a

TEI: total energy intake, ARFS: Australian recommended food score, FAVI: fruit and vegetables index, and Aus-DQI: Australian diet quality index.

**Table 2:** Weight change data (2003 to 2009) and baseline macronutrient intakes (2003) for young women in the Australian longitudinal study on women's health (ALSWH) by tertile of Australian recommended food score (ARFS).

	ARFS tertiles (Total sample *n* = 4,287)				ARFS tertiles (Valid TEI sub-sample *n* = 1,356)			
Number (*n*)	1 (*n* = 1,433)	2 (*n* = 1,426)	3 (*n* = 1,428)	*P* value (ANOVA)	1 (*n* = 402)	2 (*n* = 437)	3 (*n* = 517)	*P* value (ANOVA)
Mean ± SD	(19.7 ± 4.5)	(29.5 ± 2.0)	(39.8 ± 5.0)		(21.3 ± 3.6)	(30.1 ± 2.4)	(40.4 ± 5.1)	
ΔWeight (kg); mean ± SD	3.9 ± 8.6	3.6 ± 8.1	3.3 ± 8.4	0.2	4.0 ± 7.9	3.4 ± 7.7	2.9 ± 7.9	0.09
Baseline weight (kg); mean ± SD	68.8 ± 16.8	67.9 ± 15.5	68.2107 ± 14.9	0.16	65.7 ± 13.9	65.5 ± 14.8	65.6 ± 13.7	0.4
Follow-up weight (kg); mean ± SD	72.2 ± 18.1	71.8 ± 17.7	71.35 ± 16.3	0.44	69.7 ± 15.4	68.9 ± 16.1	69.5 ± 15.1	0.7
Energy intake (kJ/d); mean ± SD	6372.3 ± 2903.5	7091.2 ± 2905.5	7558.6 ± 2825.8	<0.0001	8819.6 ± 1339.1	8993.7 ± 392.4	9080.8 ± 1408.9	0.02
*Macronutrient and fiber intakes at baseline *								
Total fat (% energy); mean ± SD	35.2 ± 6.1	33.7 ± 5.9	32.5 ± 5.8	<0.0001	38.2 ± 5.1	37.4 ± 4.8	35.1 ± 4.9	<0.0001
Saturated fat (% energy); mean ± SD	16.4 ± 3.7	15.4 ± 3.5	14.4 ± 3.4	<0.0001	16.5 ± 3.3	15.8 ± 2.9	14.2 ± 3.0	<0.0001
Protein (% energy); mean ± SD	19.7 ± 3.4	20.5 ± 3.3	20.9 ± 3.4	<0.0001	19.2 ± 3.2	19.6 ± 2.8	20.0 ± 3.1	0.0005
Carbohydrate (% energy); mean ± SD	44.9 ± 6.9	45.5 ± 6.7	46.29 ± 6.4	<0.0001	40.9 ± 6.0	41.2 ± 5.3	42.9 ± 5.4	<0.0001
Fiber (g/d); mean ± SD	15.4 ± 6.9	18.9 ± 7.5	22.25 ± 8.0	<0.0001	20.4 ± 5.9	22.8 ± 5.7	26.1 ± 6.7	<0.0001

**Table 3:** Weight change data (2003 to 2009) and baseline macronutrient intakes (2003) for young women in the Australian longitudinal study on women's health (ALSWH) by tertile of Australian diet quality index (Aus-DQI).

	Aus-DQI tertiles (total sample *n* = 4,287)				Aus-DQI tertiles (valid TEI subsample *n* = 1,356)			
Number (*n*)	1 (*n* = 1,433)	2 (*n* = 1,426)	3 (*n* = 1,428)	*P* value (ANOVA)	1 (*n* = 402)	2 (*n* = 437)	3 (*n* = 517)	*P* value (ANOVA)
Mean ± SD	(0.6 ± 0.5)	(2.0 ± 0.0)	(3.0 ± 0.2)		(0.61 ± 0)	(1.0 ± 0.0)	(2.3 ± 0.4)	
ΔWeight (kg); mean ± SD	3.7 ± 8.5	3.7 ± 8.3	3.3 ± 7.9	0.3	3.6 ± 8.1	2.9 ± 6.9	3.5 ± 7.8	0.48
Baseline weight (kg); mean ± SD	69.1 ± 16.8	67.9 ± 14.3	66.5 ± 13.7	<0.0001	67.7 ± 15.4	63.5 ± 11.6	63.8 ± 11.8	<0.0001
Follow-up weight (kg); mean ± SD	72.3 ± 18.0	71.51 ± 16.3	69.6 ± 15.7	<0.0001	71.2 ± 16.5	66.4 ± 13.3	67.3 ± 13.9	<0.0001
Energy intake (kJ/d); mean ± SD	7658.6 ± 2839.2	7539.3 ± 2641.9	5235.7 ± 1389.9	<0.0001	9252.6 ± 1394.3	8642.1 ± 1306.7	8546.6 ± 1256.3	<0.0001
*Macronutrient and fiber intakes at baseline *								
Total fat (% energy); mean ± SD	39.1 ± 3.6	31.9 ± 3.3	28.3 ± 4.4	<0.0001	39.8 ± 3.0	35.6 ± 3.6	30.4 ± 3.7	<0.0001
Saturated fat (% energy); mean ± SD	16.5 ± 2.8	12.9 ± 2.3	11.3 ± 2.6	<0.0001	17.0 ± 2.5	14.8 ± 2.9	12.2 ± 2.3	<0.0001
Protein (% energy); mean ± SD	20.4 ± 3.4	20.65 ± 3.8	19.7 ± 3.0	<0.0001	19.8 ± 2.8	20.3 ± 3.7	18.6 ± 2.9	<0.0001
Carbohydrate (% energy); mean ± SD	38.7 ± 4.5	45.4 ± 4.0	49.8 ± 4.7	<0.0001	38.7 ± 3.9	42.2 ± 3.3	48.8 ± 4.0	<0.0001
Fiber (g/d); mean ± SD	18.7 ± 7.7	20.1 ± 9.8	17.7 ± 6.4	<0.0001	21.8 ± 5.4	23.3 ± 6.8	27.3 ± 7.4	<0.0001

**Table 4:** Weight change data (2003 to 2009) and baseline macronutrient intakes (2003) for young women in the Australian longitudinal study on women's health (ALSWH) by tertile of fruit and vegetable index (FAVI).

	FAVI tertiles (total sample *n* = 4,287)				FAVI tertiles (valid TEI subsample *n* = 1,356)			
Number (*n*)	1 (*n* = 1,433)	2 (*n* = 1,426)	3 (*n* = 1,428)	*P* value (ANOVA)	1 (*n* = 402)	2 (*n* = 437)	3 (*n* = 517)	*P* value (ANOVA)
Mean ± SD	(34.6 ± 28.0)	(83.1 ± 7.9)	(117.2 ± 18.9)		(63.5 ± 11.4)	(89.4 ± 6.5)	(120.4 ± 18.0)	
ΔWeight (kg); mean ± SD	4.4 ± 8.9	3.4 ± 7.7	3.3 ± 8.6	0.002	4.5 ± 8.6	2.9 ± 6.9	2.9 ± 7.9	0.003
Baseline weight (kg); mean ± SD	69.0 ± 17.0	68.3 ± 15.7	67.9 ± 15.1	0.1	66.1 ± 15.1	66.0 ± 14.2	65.9 ± 13.3	0.98
Follow-up weight (kg); mean ± SD	72.1 ± 17.7	71.8 ± 17.4	71.2 ± 16.9	0.3	70.6 ± 16.4	68.9 ± 15.4	68.8 ± 14.9	0.17
Energy intake (kj/d); mean ± SD	6282.7 ± 2638.8	6819.3 ± 2551.7	7602.7 ± 3296.4	<0.0001	8892.1 ± 1388.3	8924.5 ± 1333.5	9077.9 ± 1423.1	0.1
*Macronutrient and fiber intakes at baseline *								
Total fat (%); mean ± SD	35.2 ± 6.2	33.9 ± 5.8	32.9 ± 5.9	<0.0001	38.13 ± 4.9	37.12 ± 5.0	35.4 ± 5.00	<0.0001
Saturated fat (%); mean ± SD	16.4 ± 3.8	15.56 ± 3.5	14.7 ± 3.5	<0.0001	16.4 ± 3.1	15.6 ± 3.13	14.5 ± 3.1	<0.0001
Protein (%); mean ± SD	19.7 ± 3.4	20.4 ± 3.2	20.6 ± 3.5	<0.0001	19.3 ± 3.0	19.6 ± 2.9	19.8 ± 3.2	0.0491
Carbohydrate (%); mean ± SD	44.8 ± 6.7	45.4 ± 6.4	46.1 ± 6.9	<0.0001	40.7 ± 5.5	41.4 ± 5.5	42.8 ± 5.7	<0.0001
Fiber (g/d); mean ± SD	15.0 ± 6.9	17.9 ± 6.8	21.9 ± 8.4	<0.0001	20.7 ± 5.8	22.8 ± 5.9	25.8 ± 6.8	<0.0001

**Table 5:** Multiple linear regression models to predict six-year weight change in young women from the Australian longitudinal study on women's health.

Predictor: diet quality index	Model*	Tertile (versus Tertile 1)	Total sample: Δweight (kg) β coefficient (95% CI)	P value	Valid TEI subsample Δweight (kg) β coefficient (95% CI)	P value
ARFS	Crude	2	−0.32 (−0.99, 0.28)	0.29	−0.60 (−1.7, 0.46)	0.27
		3	−0.69 (−1.3, 0.08)	**0.03**	−1.14 (−2.16, −0.12)	**0.03**
	Adjusted	2	−0.16 (−0.79, 0.47)	0.63	−0.93 (−1.96, 0.09)	0.07
		3	−0.34 (−0.97, 0.30)	0.29	−1.59 (−2.63, −0.53)	**0.003**
	Final	2	−0.18 (−0.81, 0.46)	0.58	−0.95 (−2.0, 0.7)	0.07
		3	−0.38 (−1.03, 0.27)	0.25	−1.6 (−2.67, −0.56)	**0.003**

Aus-DQI	Crude	2	−0.05 (−0.71, 0.60)	0.876	−0.68 (−1.79, 0.42)	0.2
		3	−0.51 (−1.14, 0.12)	0.112	−0.10 (−1.12, 0.92)	0.8
	Adjusted	2	−0.04 (−0.71, 0.63)	0.905	−0.80 (−1.93, 0.31)	0.2
		3	−0.60 (−1.25, 0.06)	0.073	−0.46 (−1.52, 0.61)	0.4
	Final	2	−0.05 (−0.73, 0.63)	0.885	−0.81 (−1.95, 0.33)	0.2
		3	−0.62 (−1.32, 0.07)	0.078	−0.45 (1.53, 0.62)	0.4

FAVI	Crude	2	−0.96 (−1.62, −0.31)	**0.004 **	−1.60 (−2.67, −0.54)	**0.003**
		3	−1.09 (−1.75, −0.44)	**0.001 **	−1.61 (−2.62, −0.58)	**0.002**
	Adjusted	2	−0.61 (−1.28, 0.07)	0.079	−1.4 (−2.53, −0.43)	**0.006**
		3	−0.68 (−1.36, 0.00)	0.051	−1.5 (−2.59, −0.42)	**0.006**
	Final	2	−0.63 (−1.30, 0.05)	0.070	−1.5 (−2.4, −0.2)	**0.02**
		3	−0.72 (−1.42, −0.03)	**0.041 **	**−1.6 (−2.4, −0.3)**	**0.01**

*Crude model: unadjusted, Δweight: dependent variable, diet quality index: independent variable; adjusted model: adjusted for physical activity, education, number of births, area of residence, marital status, smoking, and weight at baseline; final model: the adjusted model plus total energy intake. Bold if *P* value < 0.05.
