# Supplementary material for: Diet Quality, Measured by Fruit and Vegetable Intake, Predicts Weight Change in Young Women
Source: J Obes. 2013 Aug 26;2013:525161. doi: 10.1155/2013/525161 (PMC3770048; doi:10.1155/2013/525161)
Supplement: Supplementary file 1 — Details of items, scoring methods and ARFS subscales can be viewed online in Appendxi: 1 of the Supplementary Material associated with this manuscript. [file 525161.f1.docx]

**The supplementary Material:**

**Appendix: 1** scoring methods for ARFS

|  | ARFS subscales | Range of scores and scoring items | Scoring methods |
| --- | --- | --- | --- |
| 1 | Vegetables | 22-0  1-frequncey of vegetable intake.  2-Potato cooked without fat  3.Tomato sauce/paste/dried  4. Tomato fresh or tinned  5.Peppers, Capsicum  6.Lettuce, endive, other salad greens?  7.Cucumber  8.Celery  9.Beetroot  10.Carrots  11.Cabbage, Brussels sprouts  12.Cauliflower  13.Broccoli  14.Silverbeet, Spinach  15.Peas  16.Green beans  17.Bean, or alfalfa , sprouts  18.Pumpkin  19.Onion or leeks  20.Garlic  21.Mushrooms  22.Zucchini | - Subject received one score for each item of vegetables subscale if the frequency of vegetables intake is ≥ four times per a day. - Zero score if the frequency of vegetables intake is < four times per a day. |
| 2 | Fruit | 14-0  1.Pieces fresh fruit per day  2.Fruit juice  3.Tinned, frozen fruit  4.Oranges, other citrus  5.Apples  6.Pears  7. Bananas  8.Watermelon, rockmelon, honeydew  9.Pineapple  10.Strawberries  11.Apricots  12. Peaches, nectarines  13.Mango,Paw paw  14.Avocado | About fruit intake:   - Subject received one score for each item of fruit sub-scale if a subject reported that intake of fruit pieces is ≥ two pieces per a day. - Zero score if a subject reported consumptions of fruit < two pieces per a day.   For fruit juice consumption:   - Subject received one score if reported frequency of consumptions ≥ once a week. - Zero score if a subject reported frequency of consumptions < once a week. |
| 3 | Grain | 14-0  1.High fibre white(y/N)  2.Wholemeal bread(y/N)  3.Rye bread(y/N)  4.Multi-grain bread(y/N)  5.How many slices bread per day  6.All Bran  7.Sultana Bran, FibrePlus, Branflakes  8.Weetbix, Vita Brits, Weeties  9.Rice  10.Pasta or noodles  11.Vegemite, marmite, promite  12.Cornflakes, Nutrigrain, Special K  13.Porridge  14.Muesli | - Subject received one score if reported frequency of grain consumptions ≥ once a week. - Zero score if subject reported frequency of consumptions of grains < once a week. |
| 4 | Protein | 14-0  1.Nuts  2.Peanut butter or peanut paste  3.Beef  4.Veal  5.Lamb  6.Pork  7.Chicken  8.Fish steamed, grilled, baked  9.Fish, tinned  10.Baked beans  11.Soy beans/curd/tofu  12.Soya milk  13.other beans  14.Eggs per week | - Subject received one score for each item if the frequency of protein intake is ≥ four times. - Zero score if the frequency of protein intake is < four times. |
| 5 | Dairy | 7-0  1.Reduced fat milk(y/n)Skim milk(y/N)  2.Ricotta or cottage cheese(y/N)  3.Low fat cheese(y/N)  4.How much milk per day  5.Cheese  6.Ice cream  7.Yoghurt | For milk consumption:   - Subject received one score for consuming reduce fat or skim milk if reported intake of > 500 mL per a day and received zero if consuming less than that.   For dairy product consumption:   - Subject received one score for each item of dairy product if the frequency is ≥ once a week. |
| 6 | fat | 1-0  Margarine any kind(y/n)  Polyunsaturated margarine  Monounsaturated margarine | - Subject received one score if use nil/polyunsaturated/ monounsaturated margarine and receive zero score if use other fat spread. |
| 7 | Alcohol | 2-0  1-How many glasses beer, wine, spirits  2-Beer (low alcohol),Beer (full strength), Red wine  While wine, Fortified wines, port, sherry etc, Spirits, liquers, etc | - Subject received one score for consuming ≤ 2 glasses per a day and zero if more than that. - For the frequency of alcohol intake subject received a score if consuming < once a month to a maximum 4 days per week. |

Adapted from Journal of American College of Nutrition, vol.27, No.1, 146-157(2008)
